# How Realistic Are the Scientific Assumptions of the Neuroenhancement Debate? Assessing the Pharmacological Optimism and Neuroenhancement Prevalence Hypotheses

**DOI:** 10.3389/fphar.2018.00003

**Published:** 2018-01-22

**Authors:** Stephan Schleim, Boris B. Quednow

**Affiliations:** ^1^Theory and History of Psychology, Faculty of Behavioral and Social Sciences, Heymans Institute for Psychological Research, University of Groningen, Groningen, Netherlands; ^2^Experimental and Clinical Pharmacopsychology, Department of Psychiatry, Psychotherapy and Psychosomatics, Psychiatric Hospital, University of Zurich, Zurich, Switzerland; ^3^Neuroscience Center Zurich, University of Zurich and ETH Zurich, Zurich, Switzerland

**Keywords:** smart drugs, study drugs, cognitive enhancement, neuroenhancement, stimulants, modafinil, methylphenidate, amphetamine

## Abstract

Since two decades, neuroenhancement is a major topic in neuroethics and still receives much attention in the scholarly literature as well as in public media. In contrast to high hopes at the beginning of the “Decade of the Brain” in the United States and Europe that we subsume under the “pharmacological optimism hypothesis,” recent evidence from clinical neuroscience suggests that developing drugs that make healthy people smarter is even more difficult than finding new treatments for patients with mental disorders. However, cognitive enhancing drugs even for patients with impaired intellectual performance have not been successfully developed yet and new drugs that might have a disruptive impact on this field are unlikely to be developed in the near future. Additionally, we discuss theoretical, empirical, and historical evidence to assess whether cognitive enhancement of the healthy is common or even epidemic and if its application will further increase in the near future, as suggested by the “neuroenhancement prevalence hypothesis.” Reports, surveys, and reviews from the 1930s until today indicate that psychopharmacological neuroenhancement is a fact but less common than often stated, particularly in the public media. Non-medical use of psychostimulants for the purpose of cognitive enhancement exists since at least 80 years and it might actually have been more common in the past than today. Therefore, we conclude that the pharmacological optimism hypothesis and neuroenhancement prevalence hypotheses have to be rejected and argue that the neuroenhancement debate should take the available evidence more into account.

## Introduction

**FIGURE 1:**
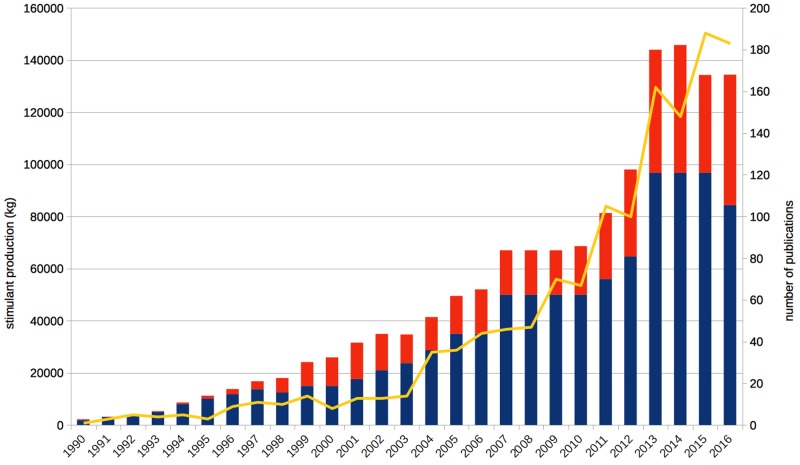
Annual publications on enhancement have grown steadily since the early 1990s (yellow line, right axis), in parallel to the annual production quotas of methylphenidate (blue bars) and amphetamine (red bars; both left axis). “Cognitive enhancement” is by far the most common term with 1,065 hits for the whole period, followed by “neuroenhancement,” which was mentioned first in 2004, achieving a total of 180 hits so far. Based on data from the ISI Web of Science topic search for cognitive, affective, mood enhancement, and neuroenhancement as well as the US Drug Enforcement Agency and the US Federal Register.

Since the late 1990s, scientists, ethicists, and legal scholars debate the issue of neuroenhancement – the improvement of healthy people’s cognitive functioning on the neural level, for example by psychopharmacological means (Whitehouse et al., 1997; Farah et al., 2004). Other possible strategies, such as brain stimulation or genetic modification, are presently being investigated and discussed as well (e.g., Hamilton et al., 2011). However, because of the higher prevalence and longer history of psychopharmacological approaches, we focus on stimulant drugs in the present paper, particularly methylphenidate, modafinil, and amphetamine. The scholarly interest in neuroenhancement has steadily increased since the 1990s, as reflected by the number of annual publications (**Figure 1**). It is also a revenant topic in the media communication about brain research: The broad public or at least the decision-makers of the popular press address “brain optimization” even more frequently than mental disorders (O’Connor et al., 2012). The vast majority of such reports describes neuroenhancement as common, increasing, or both (Partridge et al., 2011), but we also noted many scientific publications doing so (Quednow, 2010; Schleim, 2010; Schleim and Quednow, 2017).

The sustained enthusiasm about and interest in pharmacological neuroenhancement is frequently based on three assumptions, (1) that intellectual performance can putatively be improved by drugs, (2) that pharmacological neuroenhancement is already done commonly by healthy people, and (3) that it will be used increasingly in the future. If neuroenhancement were impossible, at least in the short- to mid-term, or if almost nobody used drugs for such purposes, the debate would probably lose much of its public relevance, although there would be still other ethical issues for discussion. We would like to coin the first assumption the “pharmacological optimism hypothesis” and summarize the two latter ones to the “neuroenhancement prevalence hypothesis.” With theoretical considerations, reviewing recent surveys on prevalence of neuroenhancement including historical evidence from Germany, Switzerland, the Netherlands, and the United States, we will assess both hypotheses in this paper to provide a better evidence base for the ethical neuroenhancement debate.

## The Pharmacological Optimism Hypothesis

Essential support for our arguments is coming from the so-called funding crisis in psychopharmacology that arose in ca. 2010^1^ and from past and current reports on the consumption patterns of psychostimulant users and the low prevalence of their use as neuroenhancers. Optimistic expectations to find better treatments for neurodegenerative or psychiatric disorders were central to the “Decade of the Brain” proclaimed by the U.S. Government and the European Commission (Bush, 1990; Pandolfi, 1993), but also to influential political initiatives that prioritized funding of that research area. The German manifesto on the future of brain research published by eleven influential neuroscientists (Monyer et al., 2004) and the Human Brain Project^2^, funded by the European Research council since 2013, are further examples for the confidence regarding new treatments developed by clinical neuroscience. Similarly, a major aim of the fifth edition of the Diagnostic and Statistics Manual (DSM-5) of the American Psychiatric Association published in 2013 was the discovery of neuroscientific biomarkers that are reliable targets particularly in the brain or genome for diagnosis and treatment of psychiatric disorders (Kupfer et al., 2002; Hyman, 2007). In spite of these efforts and an unprecedented increase in scientific publications and knowledge, the high expectations in terms of translations to clinical applications were not met yet (Schleim and Roiser, 2009; Schleim, 2014a; Frisch, 2016). The failure to discover even a single reliable biomarker for any of the hundreds of DSM-5 classifications lead to the introduction of a new research paradigm, the Research Domain Criteria (RDoC), whose scientific superiority remains unclear at the present moment (Kirmayer and Crafa, 2014).

In accordance with the high expectations of the 1990s and early 2000s regarding clinical neuroscience, the neuroenhancement literature was optimistic that new drugs for dementia or attention disorders could also be used for improving cognitive functioning in healthy people (Whitehouse et al., 1997; Farah et al., 2004). In contrast to these hopes, the funding crisis of psychopharmacology became evident around 2010: On the one hand, governmental changes in the funding structures of many countries made scientists in this area more dependent on collaborations with the pharmaceutical industry (Stanford, 2008; Hendrie, 2010). On the other hand, a lot of pharmaceutical companies closed their respective laboratories and rather invested in other fields because of the lack of successes of newly developed compounds resulting in high business risks regarding the introduction of new medications (Miller, 2010; Nutt and Goodwin, 2011; van Gerven and Cohen, 2011).

From this perspective it is not surprising that a major part of the psychopharmacological neuroenhancement literature (Smith and Farah, 2011; Weyandt et al., 2013; Busardo et al., 2016) covers well-known stimulant drugs that have been discovered a long time ago, like amphetamine, already synthesized at the end of the 19th century, methylphenidate, a discovery of the 1940s, and modafinil, synthesized in the 1970s. All of these drugs were or are still prescribed for some psychiatric indications with some differences between countries related to, e.g., the substances’ abuse potential.^3^ However, that the molecules have been known and investigated for a long time does not mean that they do not pose scientific challenges any more. Amphetamine, by far the oldest of the three compounds, still keeps scientists busy who want to understand the precise mechanism of action in the animal and human brain (Sulzer et al., 2005). A recent Cochrane meta-analysis of the available clinical studies on amphetamine for attention-deficit/hyperactivity disorder (ADHD) treatment found that most trials were at a high risk of bias, provided low to very low quality evidence, and should be longer in duration to learn more about long-term side effects of the treatment (Punja et al., 2016). The latter point is of particular interest when neuroenhancement in the healthy is performed not just for a particular event, like an exam period, but to increase performance continuously.

These observations demonstrate that psychopharmacological research is complex, challenging, and difficult even in the case of neurological and psychiatric disorders, where the treatment outcome is clear, such as a reduction of symptom severity associated with an improvement of social and occupational functioning. Moreover, the ethical issue of intervening in the brain chemistry is justified by patients’ suffering, but might be disputable in healthy people. In the case of cognitive-emotional disturbances in mental disorders, clinically validated and reliable neuropsychological tests are available to measure the treatment’s outcome; however, most of the so far tested substances have very limited effects on disturbed cognitive functions in neurological and psychiatric patient populations (Dekkers and Rikkert, 2007; Chou et al., 2012; de Jongh, 2017). In contrast to these clinical standards, it is much less clear what the outcome of neuroenhancement in the healthy would be and how it could be measured. Employing the same neuropsychological tests as in clinical studies would carry the risk of the fallacy that what helps patients must also help the healthy (Schleim, 2014b). That this reasoning is not necessarily true can be shown with many examples, such as insulin which is essential for patients with diabetes but would not help and even harm people without the disease. According to an influential definition, human enhancement is “[a]ny change in the biology or psychology of a person which increases the chances of leading a good life in the relevant set of circumstances” (Savulescu et al., 2011). This could be virtually everything and the meaning of “a good life” can be expected to strongly vary across people (Schleim, 2014b). Either way, the evidence is still low that so far discussed drugs in fact broadly enhance cognitive performance in the healthy (de Jongh et al., 2008; Quednow, 2010; Wood et al., 2014). We assume that if the situation of psychopharmacology were more positive, with a high availability of clinically validated new treatments for neurological and mental disorders, optimism concerning psychopharmacological neuroenhancement might be justified. However, in the present situation we have to reject the pharmacological optimism hypothesis, which does not amount to sheer pessimism but rather a pharmacological realism considering the evidence discussed above (Schleim and Quednow, 2017).

## The Neuroenhancement Prevalence Hypothesis

As already summarized in the introduction, public media often describe ways to improve one’s brain and pharmacological neuroenhancement as common, increasing, or both (Partridge et al., 2011; O’Connor et al., 2012). A detailed analysis has shown that scientific sources are often quoted as evidence for such statements (Partridge et al., 2011), which is in line with our own perception of the scholarly literature. At first glance, stimulant production figures seem to support this finding: The aggregate production of methylphenidate and amphetamine combined in the United States was about 100.000 kg in the 1990s, 500.000 kg in the first decade of the 2000s and already more than 800.000 kg in the 7 years from 2010 to 2016 (**Figure 1**). Thus, the amount deemed sufficient during a *whole decade* in the 1990s is surpassed *annually* since 2013 with 134.000–146.000 kg of just these two psychostimulants produced per year. According to the neuroenhancement prevalence hypothesis, one would expect a similar increase of the prevalence of non-medical prescription stimulant consumption, for example, on college or university campuses. In contrast, this is not what the data show: Although the reported prevalence rates vary from nearly 0% to more than 30% in individual studies, the most recent and most comprehensive reviews found that the methodologically best studies (e.g., comprising the largest and most representative samples) frequently reported prevalence rates well below 10% (Smith and Farah, 2011; Weyandt et al., 2013). Importantly, consumption generally operationalized as non-medical use often included other motives beyond cognitive performance enhancement, such as recreational/lifestyle use in order to have fun, to party, or to lose weight, and often referred to lifetime or last year prevalence, which does not provide more information than that users have consumed such substances at least once during long periods of time. For example, one study, which reported a lifetime prevalence of 16.2%, found that only 15.5% of this subsample, or 2.5% of the original sample, were regular users who took prescription stimulants non-medically at least two or three times per week (White et al., 2006).

One of the first surveys in Germany showed that the lifetime prevalence of the use of neuroenhancers was only 1.3% in a large sample of pupils and students (Franke et al., 2011). A more recent nation-wide survey among students reported low prevalence rates for specific neuroenhancement use of prescription drugs (1.7%, methylphenidate, modafinil, or beta blockers; at least “sometimes”) or illicit drugs (1.3%, e.g., cocaine) in the Netherlands (Schelle et al., 2015). Similarly, a nation-wide survey among university students in the United Kingdom and Ireland reported that 0.8, 3.4, or 0.3% were current users of methylphenidate, modafinil, or amphetamine, respectively, for the purpose of neuroenhancement (Singh et al., 2014). Finally, also in Swiss students the lifetime prevalence rates of using methylphenidate (3.7%), modafinil (0.3%), amphetamine (0.4%), and cocaine (0.2%) exclusively for cognitive enhancement purposes were rather low and clearly non-epidemic (Maier et al., 2013).

Of course it is debatable how high the percentage of consumers needs to be to properly speak of a “common” or even “epidemic” use. However, there is currently no evidence, to our knowledge, that the numbers have really been increasing in the past 20 years. The situation is further complicated by different inclusion criteria (e.g., general non-medical vs. specific neuroenhancement use) and outcome measures (e.g., once-in-a-lifetime vs. regular use) of the studies. By contrast, the evidence more likely suggests that many of the consumers responding positively in the surveys are young people trying out prescription stimulants for neuroenhancement or other non-medical and recreational use just once or only a few times – and then stop doing so (Sussman et al., 2006; Schleim and Quednow, 2017). It is well known that college students have a high likelihood of experimenting with different kinds of illicit drugs and dangerous behaviors (Dennhardt and Murphy, 2013) but that they usually stop this behavior when they leave the college (Johnston et al., 2005). Summarizing all of the above, it is thus highly likely that the increase in psychostimulant production in the United States (**Figure 1**) and many other countries largely reflects an increase in medical use induced by a change in prescription patterns of physicians as it was shown for the increase of methylphenidate production during the 1990s and 2000s in Germany (Ferber et al., 2001; Schubert et al., 2010). However, such prescriptions are usually excluded in surveys on the prevalence of non-medical stimulant use, in accordance with the basic assumption of the ethical neuroenhancement debate that treatment has to be distinguished from enhancement (Council on Bioethics, 2003).

In fact, the frequency of diagnosing ADHD for which methylphenidate and, in some countries, also amphetamine are commonly prescribed, has been increased since the 1970s and is estimated to have reached 7.2% of children and adolescents presently on the basis of a large meta-analysis (Thomas et al., 2015). The rate of children and adolescents prescribed with ADHD medication has increased accordingly, approaching 4% in the Netherlands and the United States, 2% in Denmark and Germany, but remaining at only 0.5% in the United Kingdom (Bachmann et al., 2017), partially explaining the increase of stimulant production seen in **Figure 1**. In summary, these data make plausible why we only see an increase of prescription stimulants in production quotas, but not in surveys investigating the prevalence of neuroenhancement. Given the high availability of the drugs because of medical prescriptions, one might have expected even higher prevalence rates of non-medical use. For the time being, we consider the presented arguments as sufficient justification to reject the neuroenhancement prevalence hypothesis.

## History of Neuroenhancement

In addition to this evidence concerning the present situation, we can also present historical sources to support our arguments even further. Rasmussen (2008) already has drawn insightful parallels between medical use of psychostimulants in the early 2000s and before the 1970s. We identified publications documenting the use of amphetamine as study drugs, thus non-medically as neuroenhancement, as early as in the 1930s. For example, an editorial in the *Journal of the American Medical Association* of 1937 stated that “…*this information* [about the psychological outcomes of an amphetamine experiment at the University of Minnesota] *was disseminated to the student body by word of mouth and the drug has been and still is being obtained by the students from drug stores for the purpose of avoiding sleep and fatigue when preparing for examinations*” (Goodman and Gilman, 1937). A follow-up editorial a year later contained a general warning about the substance and stated that “*news that it could be obtained for keeping one awake while ‘cramming’ for final examinations spread from campus to campus*” (Anon, 1938). The Dutch physician Meerloo (1937) wrote that three of his patients, all of them students who had taken amphetamine to study longer at night, suffered from unwanted side-effects or test anxiety. In Germany, an experiment carried out in September 1938 with students at the Military Academy of Berlin is documented in which placebo, caffeine,and amphetamine were compared when students learned under conditions of sleep deprivation (Ohler, 2015).

Psychostimulant use for neuroenhancement purposes occurred even after the “War on Drugs” was proclaimed in the early 1970s, which introduced harsh punishments for amphetamine usage: “*The occasional use of amphetamine to remain alert or enhance one’s performance is widespread. Students cramming for exams, drivers on extended nonstop trips, athletes attempting to excel, and military personnel on prolonged operations are some of the groups involved*” (Cohen, 1975). We documented elsewhere that surveys carried out in the 1960s, 1970s, and 1980s found similar and in some cases even higher prevalence rates for stimulant consumption than those discussed above, particularly for amphetamine, including instrumental use to stay awake and/or to study, thus which would be called “neuroenhancement” nowadays (Schleim and Quednow, 2017). The combined historical evidence from the 1930s to the 1980s makes our case for the rejection of the neuroenhancement prevalence hypothesis even stronger.

## Conclusion

Psychopharmacological neuroenhancement – or cognitive enhancement – exists at least for more than 80 years. Only the concept is new; and the surge in related publications documented in **Figure 1**. But even before the contemporary debate, some scholars raised ethical and theoretical issues concerning stimulant consumption long before so-called “neuroethics” came into existence. Smith and Blachly (1966) already asked, whether subjects really perform better or just perceived themselves so, in how far socioeconomic status affects consumption, why so many students rather consume the drugs occasionally than regularly, whether medical students are at a higher risk, or to what extent the practice is influenced by the pharmaceutical industry. Unfortunately for those patients who are waiting for better treatments, psychopharmacological research turned out to be more difficult than suggested during the very optimistic 1990s and early 2000s in which also the present ethical neuroenhancement debate has its roots. We have argued that if it is even challenging to develop new treatments, then finding drugs which are suitable for improving cognitive functioning of the healthy with acceptable long-term side-effects is even more difficult, for theoretical, pharmacological, and ethical reasons. Therefore we clearly reject the pharmacological optimism and neuroenhancement prevalence hypotheses as explained above.

The neuroenhancement debate has been called a “myth” (Zohny, 2015), a “bubble” (Lucke et al., 2011), and a “phantom debate” (Quednow, 2010) independently by various authors. From our perspective, the already common phenomenon of students’ drug use was re-framed as a new ethical and epidemiological problem in academic discourses, making use of exaggerated promises and prevalence rates. We do not say that scientists, ethicists, or legal scholars should stop debating neuroenhancement, but that this debate should rest on correct theoretical, empirical, and historical evidence in order to avoid unrealistic expectations in the general public (Forlini and Racine, 2009; Forlini and Hall, 2016). Other authors criticized the repetitiveness of this debate since the 1990s (Brenninkmeijer and Zwart, 2017). Furthermore, as psychopharmacology is in a funding crisis, relocation of resources for improving the already healthy probably would imply further negative consequences for many patients. Meanwhile, if the modification of biopsychological factors to improve people’s chances of leading a good life (Savulescu et al., 2011) turns out to be more difficult than expected, we propose a shift to the environmental and social factors affecting people’s well-being as an alternative (Schleim, 2014b).

## Author Contributions

All authors listed have made a substantial, direct and intellectual contribution to the work, and approved it for publication.

## Conflict of Interest Statement

The authors declare that the research was conducted in the absence of any commercial or financial relationships that could be construed as a potential conflict of interest.

## Footnotes

1 For example, *nature news* featured “Psychopharmacology in crisis” in June 2011 (http://www.nature.com/news/2011/110614/full/news.2011.367.html). We will address more scholarly sources below.

2 https://www.humanbrainproject.eu/

3 For a comparison of legislations within Europe, see information provided by the European Monitoring Centre for Drugs and Drug Addiction http://www.emcdda.europa.eu/html.cfm/index146601EN.html
